# Supplementary figures and images for: Tumor-infiltrating nerves functionally alter brain circuits and modulate behavior in a mouse model of head-and-neck cancer
Source: eLife. 2024 Sep 20;13:RP97916. doi: 10.7554/eLife.97916 (PMC11415076; doi:10.7554/eLife.97916)

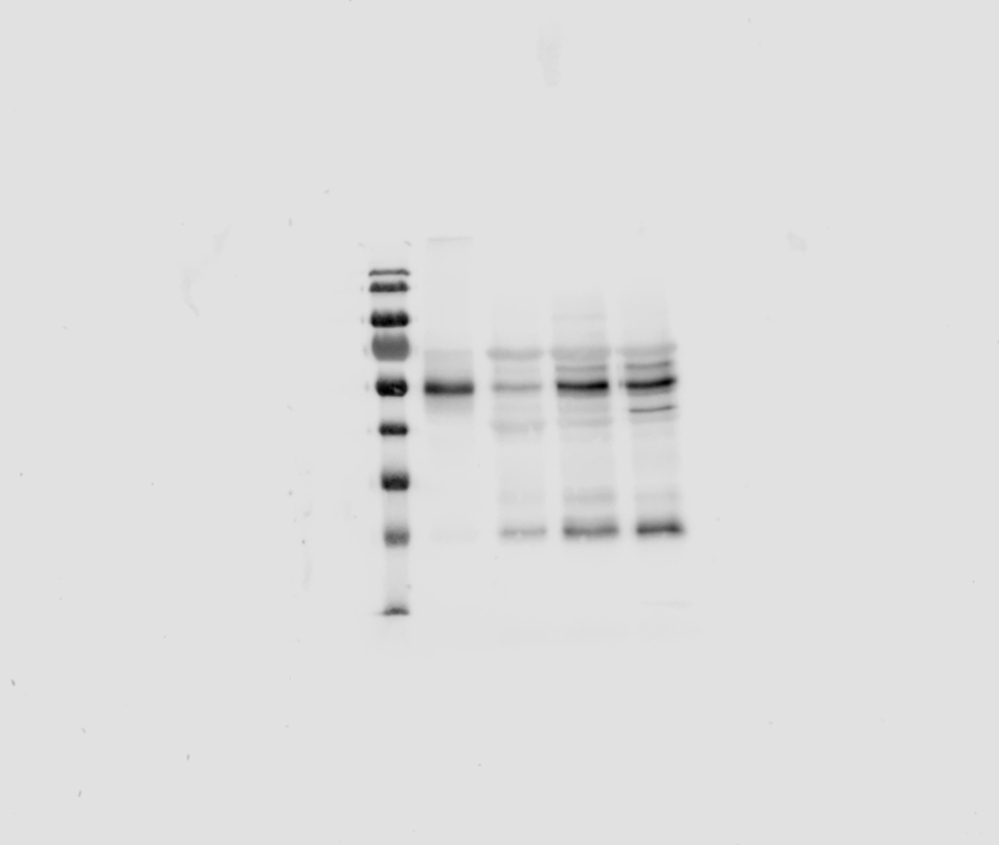

Supplement: Figure 1—source data 1. [file elife-97916-fig1-data1.zip › Raw unedited gel for Figure 1a.jpg]

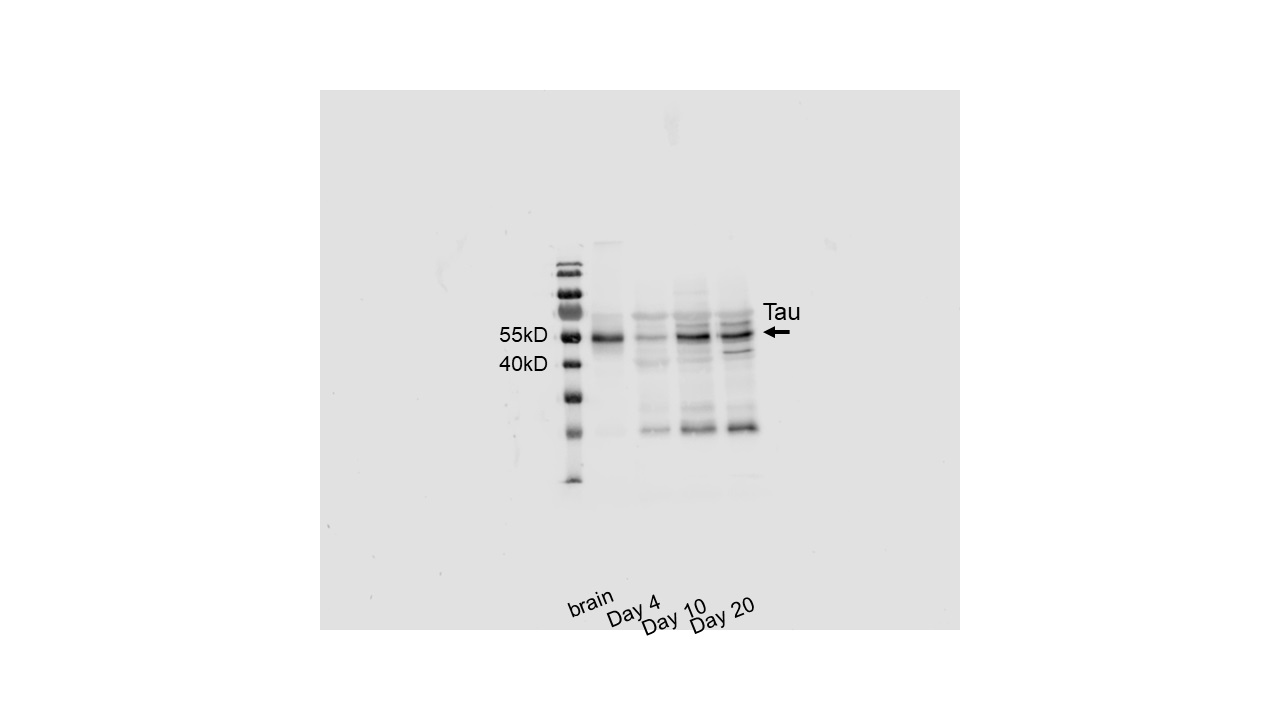

Supplement: Figure 1—source data 2. [file elife-97916-fig1-data2.zip › Uncropped and labeled gel for Figure 1a.jpg]

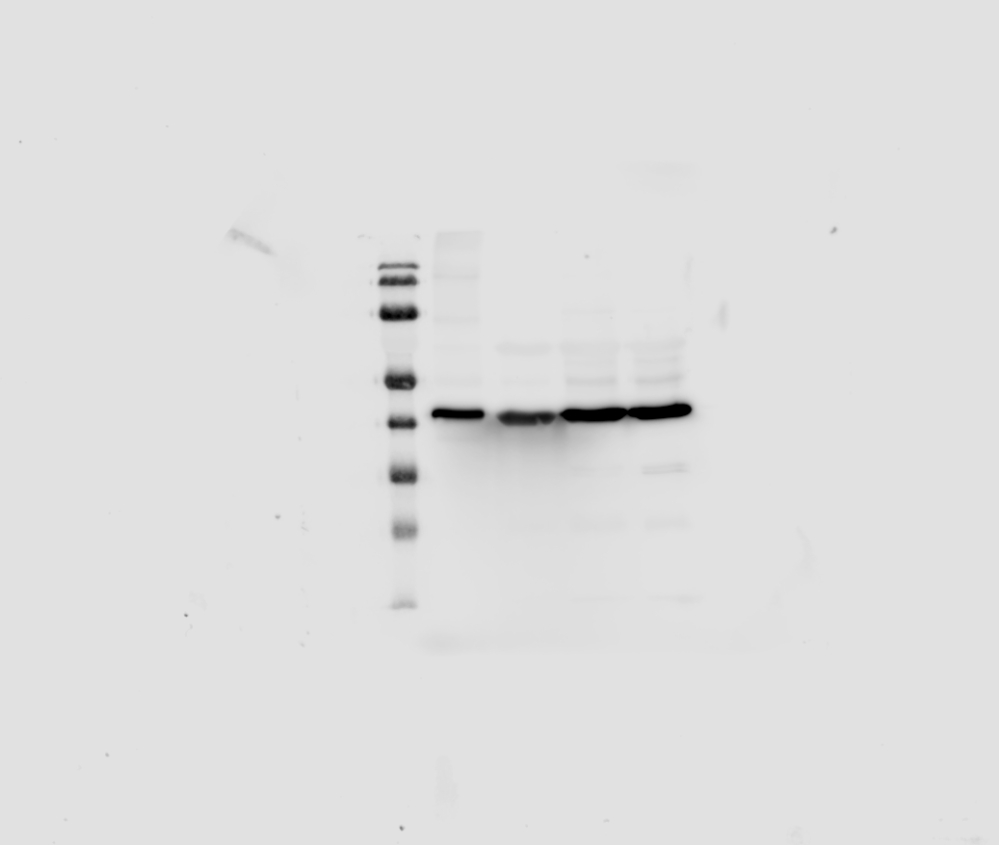

Supplement: Figure 1—source data 3. [file elife-97916-fig1-data3.zip › Raw unedited gel for Figure 1a.jpg]

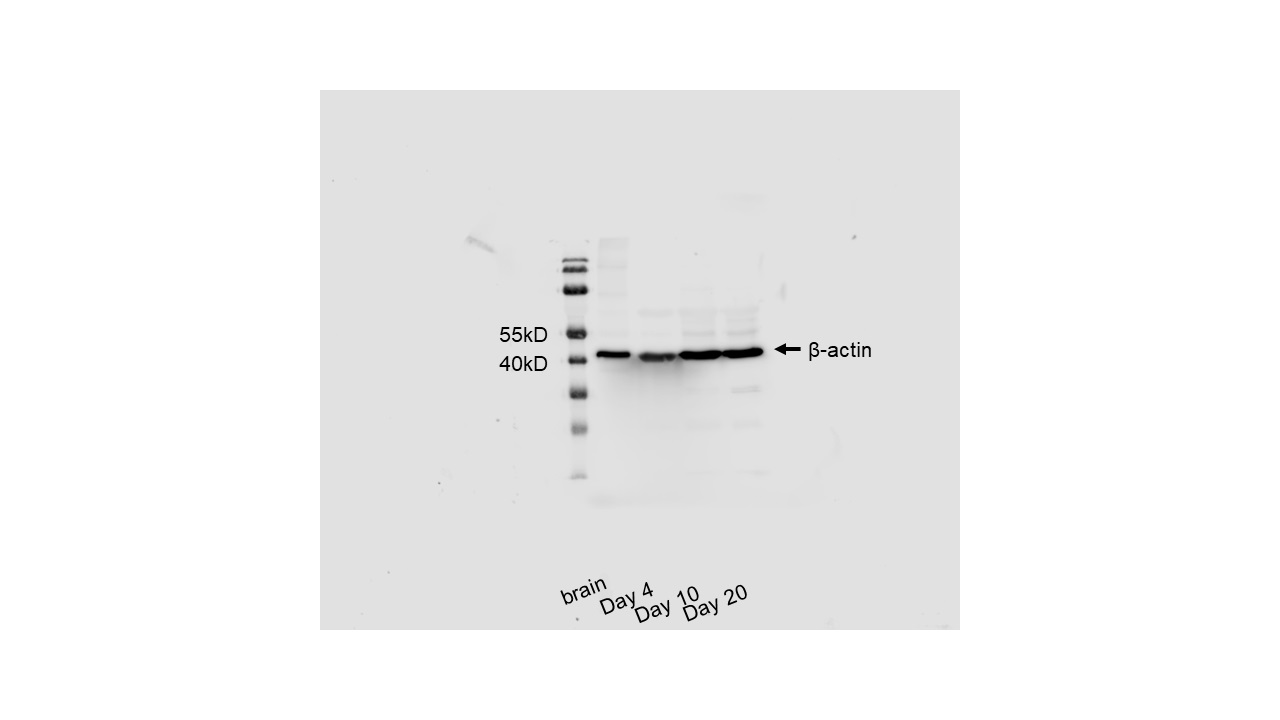

Supplement: Figure 1—source data 4. [file elife-97916-fig1-data4.zip › Uncropped and labeled gel for Figure 1a.jpg]

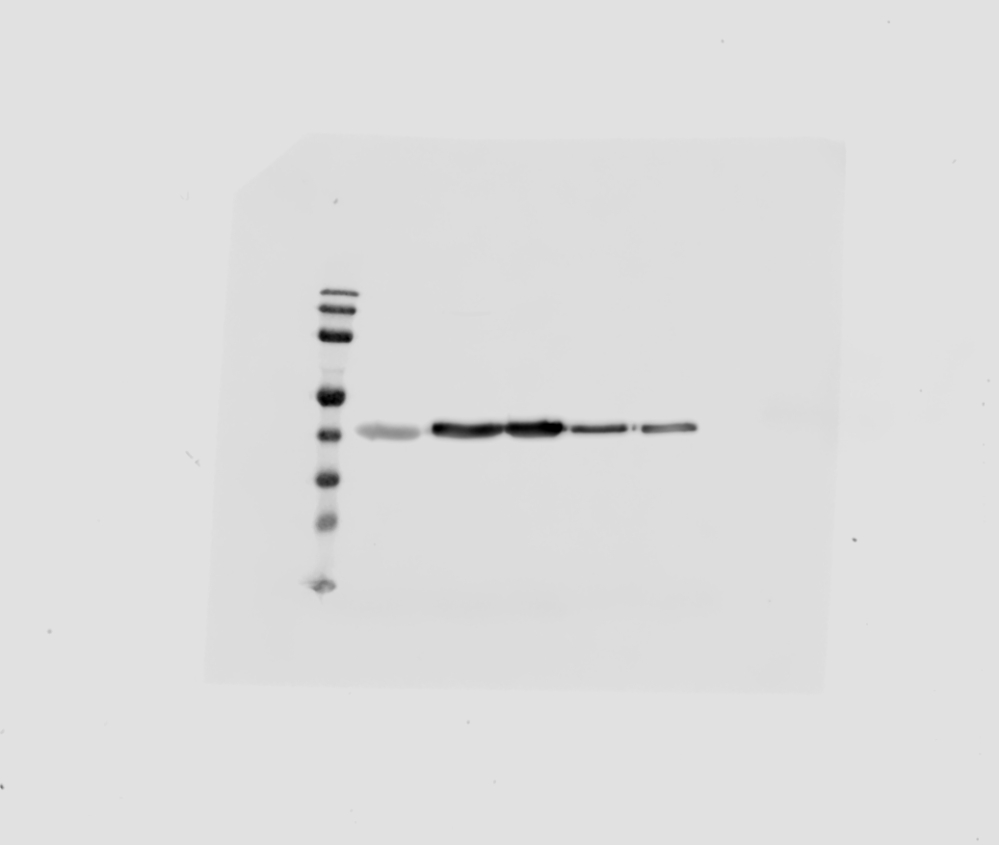

Supplement: Figure 1—source data 5. [file elife-97916-fig1-data5.zip › Raw unedited gel for Figure 1b.jpg]

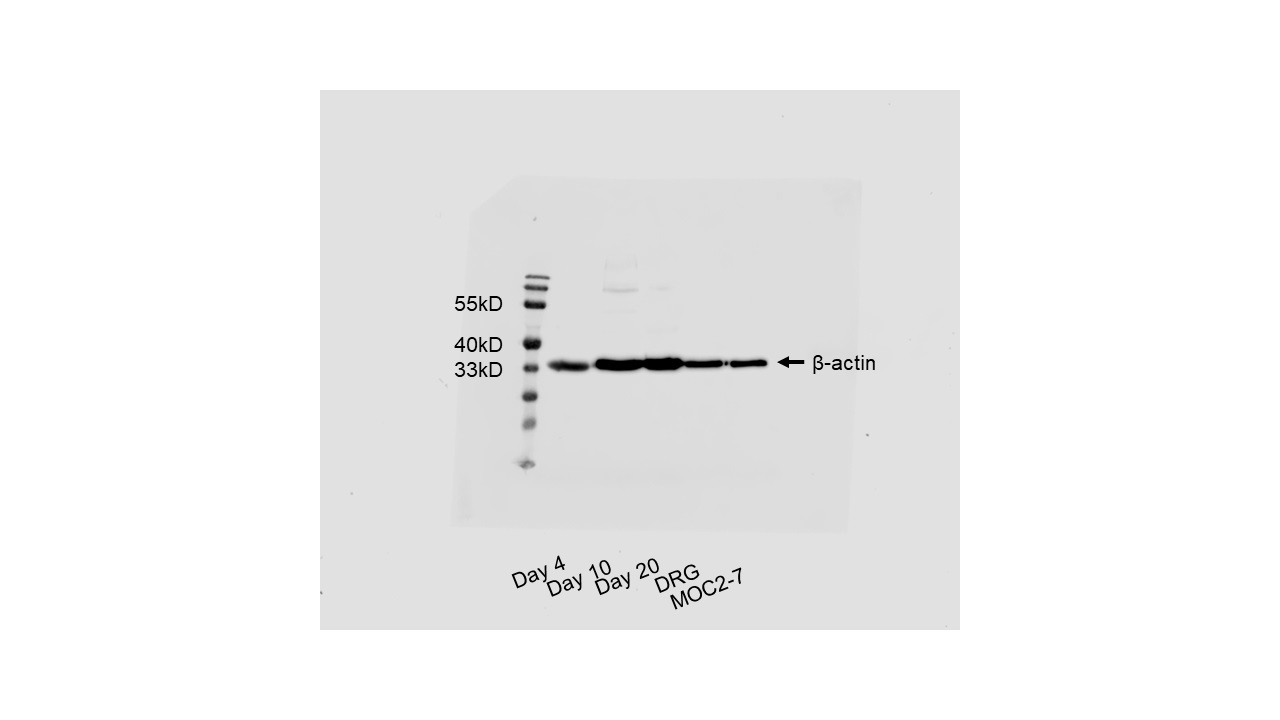

Supplement: Figure 1—source data 6. [file elife-97916-fig1-data6.zip › Uncropped and labeled gel for Figure 1b.jpg]

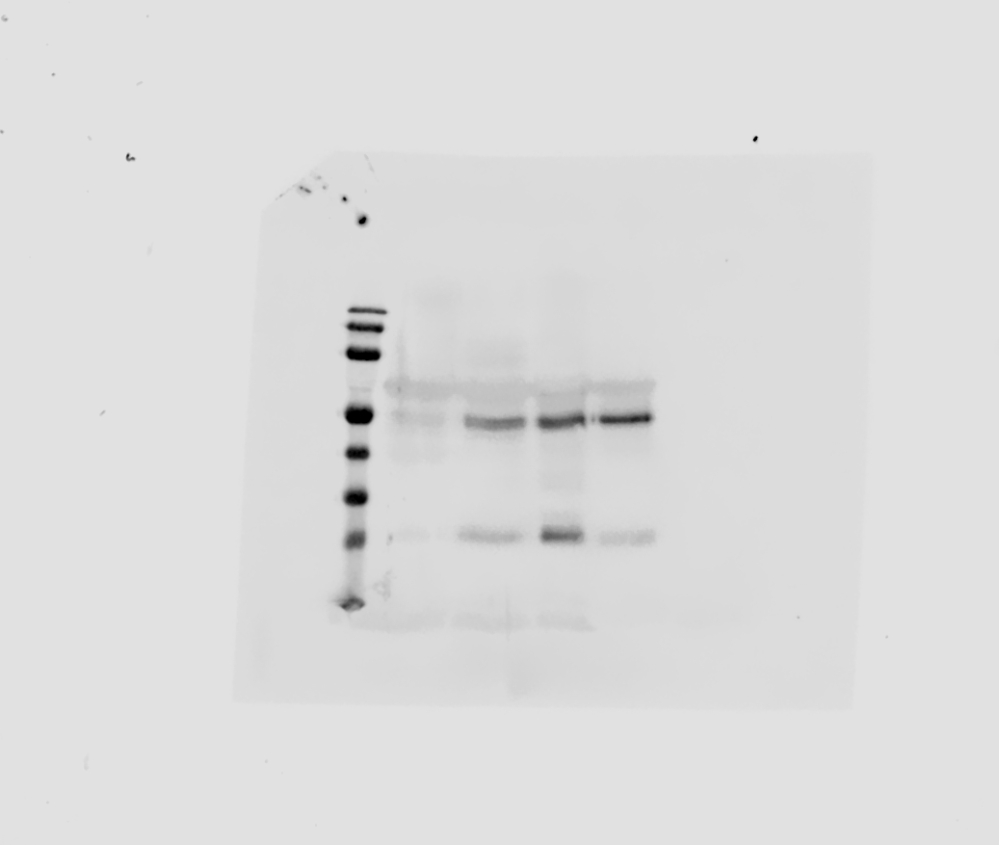

Supplement: Figure 1—source data 7. [file elife-97916-fig1-data7.zip › Raw unedited gel for Figure 1b.jpg]

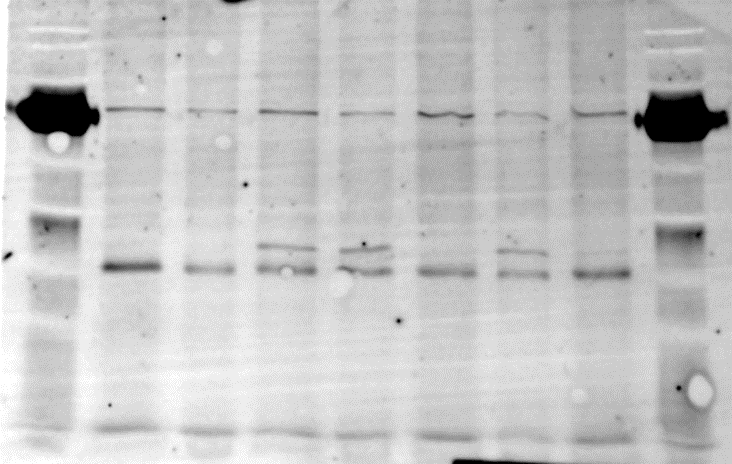

Supplement: Figure 2—source data 1. [file elife-97916-fig2-data1.zip › Raw unedited gel for Figure 2g.docx]

pTRPV1


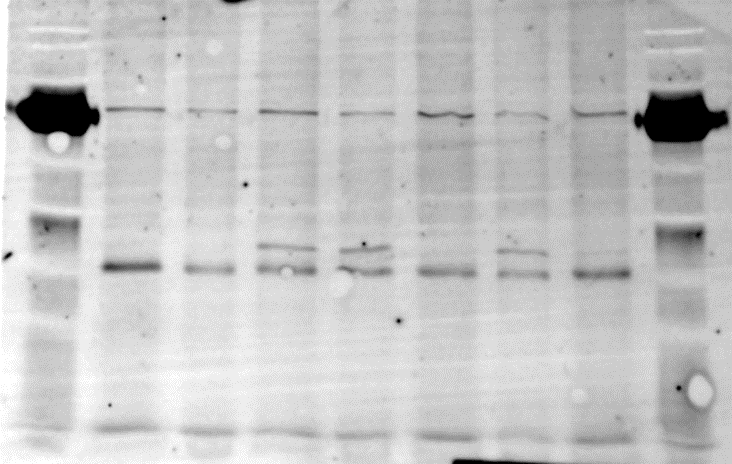


170kD

130kD

95kD

34kD

26kD

72kD

55kD

43kD

N

N

N

T

T

T

T

B.

Supplement: Figure 2—source data 2. [file elife-97916-fig2-data2.zip › Uncropped and labelled gel for Figure 2g.docx]

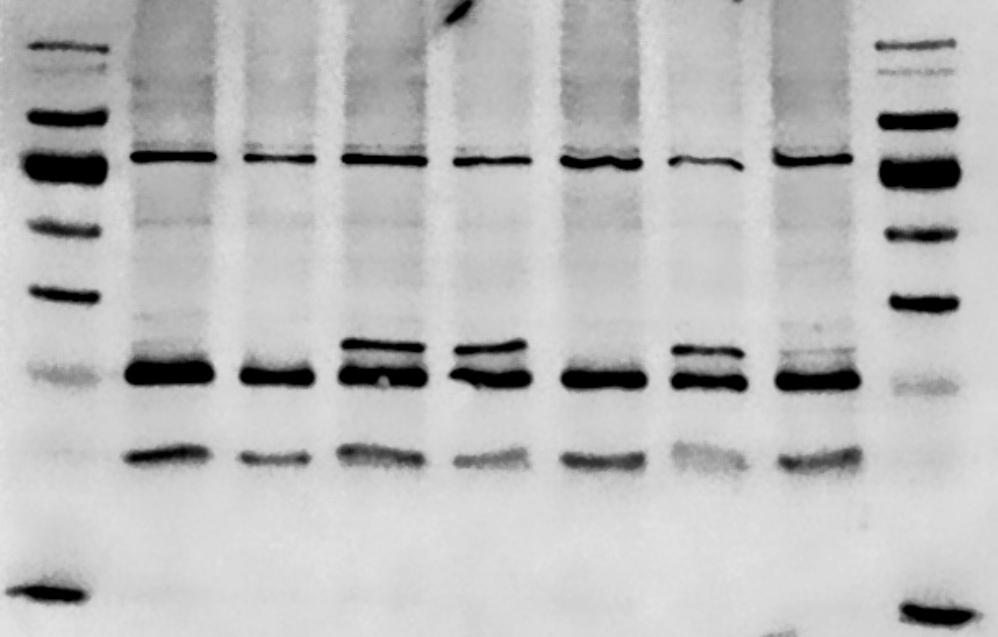

Supplement: Figure 2—source data 3. [file elife-97916-fig2-data3.zip › Raw unedited gel for Figure 2g.docx]

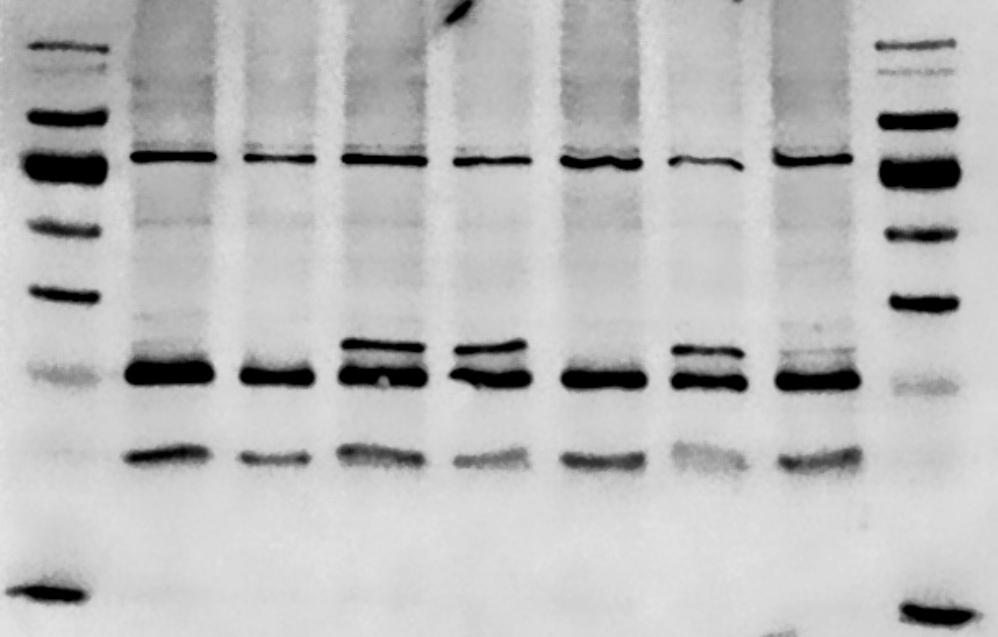


170kD

130kD

95kD

34kD

26kD

72kD

55kD

43kD

Sigma1-R

N

N

N

T

T

T

T

Supplement: Figure 2—source data 4. [file elife-97916-fig2-data4.zip › Uncropped and labelled gel for Figure 2g.docx]

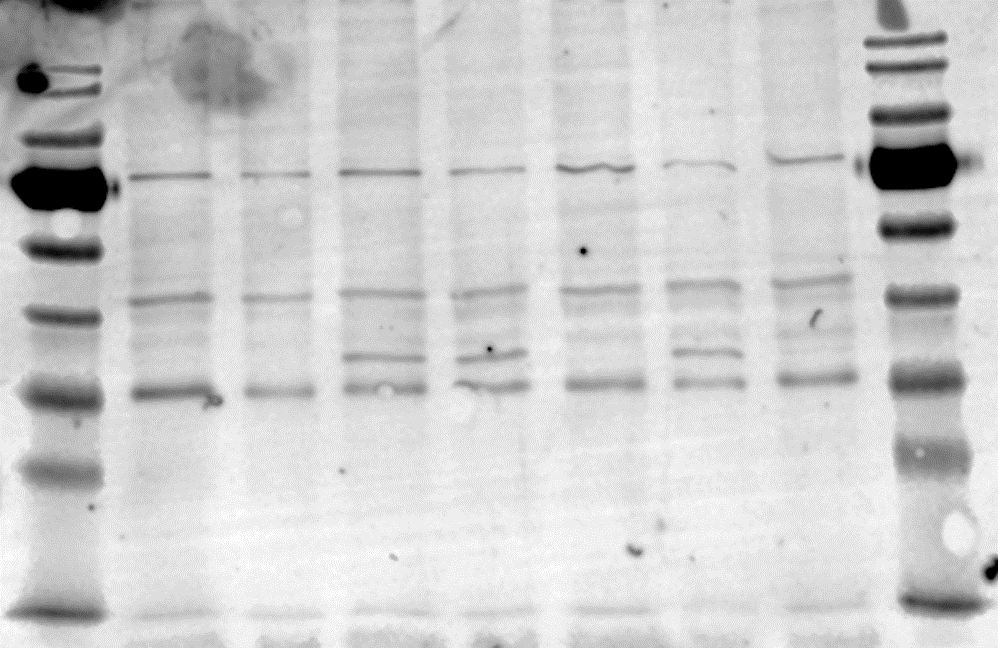

Supplement: Figure 2—source data 5. [file elife-97916-fig2-data5.zip › Raw unedited gel for Figure 2g.docx]

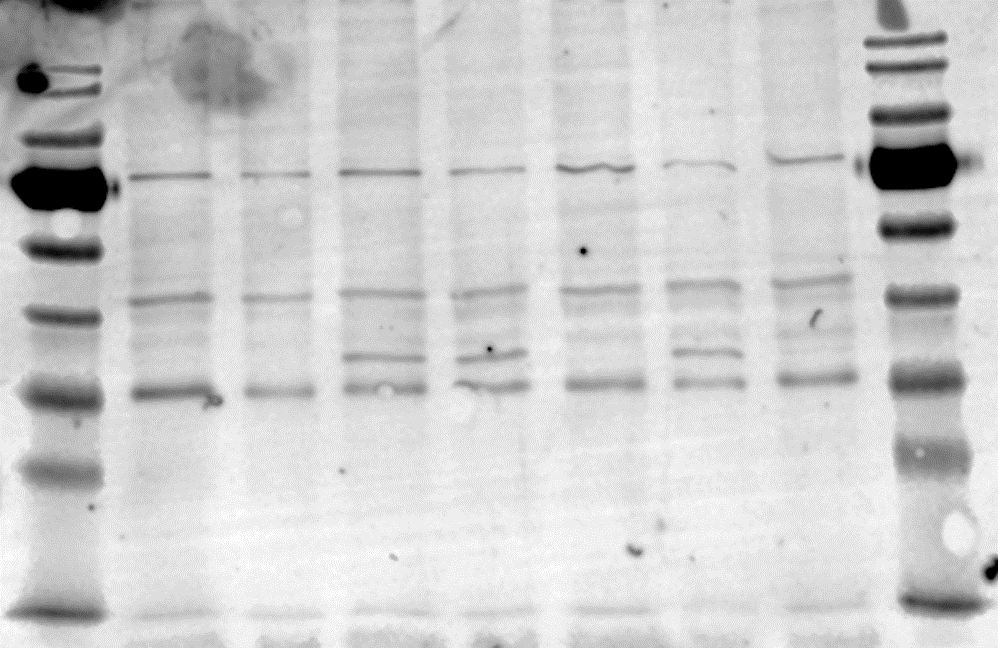


b-actin

170kD

130kD

95kD

34kD

26kD

72kD

55kD

43kD

N

N

N

T

T

T

T

Supplement: Figure 2—source data 6. [file elife-97916-fig2-data6.zip › Uncropped and labelled gel for Figure 2g.docx]
