# Supplementary material for: Tumor-infiltrating nerves functionally alter brain circuits and modulate behavior in a mouse model of head-and-neck cancer
Source: eLife. 2024 Sep 20;13:RP97916. doi: 10.7554/eLife.97916 (PMC11415076; doi:10.7554/eLife.97916)
Supplement: Supplementary file 1. [file elife-97916-supp1.docx]

| *Adra1a*  *Adra1d*  *Adra2a*  *Adrb2*  *Adrb3*  *Akr1b1*  *Aldh5a1*  *Als2* | *Bdnf*  *Btd*  *Chmp2b*  *Chrm1*  *Chrm4*  *Chrm5*  *Chrna3*  *Chrna4* | *Chrna5*  *Chrna6*  *Chrna7*  *Chrne*  *Comt*  *Dbh*  *Dhfr*  *Drd1* | *Drd2*  *Drd4*  *Drd5*  *Fig4*  *Fus*  *G6pd*  *Gabbr1*  *Gabbr2* | *Gabra2*  *Gabra4*  *Gabra5*  *Gabrb1*  *Gabrb3*  *Gabre*  *Gabrg1*  *Gabrg2* | *Gabrg3*  *Gabrg*  *Gabrr2*  *Gch1*  *Ch1*  *Gria1*  *Gria2*  *Gria3* | *Grik1*  *Grik2*  *Grik4*  *Grik5*  *Grin2a*  *Grin2b*  *Grin2c*  *Grm1* |
| --- | --- | --- | --- | --- | --- | --- |
| *Grm3*  *Grm4*  *Grm6*  *Grm7*  *Htr1a*  *Htr1b*  *Htr1d*  *Htr1f* | *Htr2a*  *Htr2c*  *Htr3a*  *Htr4*  *Htr7*  *Llgl1*  *Lpl*  *Maoa* | *Optn*  *Otc*  *Ptc*  *Qdpr*  *Rab10*  *Slc6a2*  *Slc6a3*  *Slc6a4* | *Snap25*  *Sod1*  *Spr*  *Tardnp*  *Th*  *Tph1*  *Vapb*  *Vcp* | *Actb*  *Gapdh*  *Ldha*  *Nono*  *Ppih*  *Gdc*  *Ppc*  *Ntc* |  |  |
